# Micronutrient intakes and potential inadequacies of community-dwelling older adults: a systematic review

**DOI:** 10.1017/S0007114515000203

**Published:** 2015-03-30

**Authors:** Sovianne ter Borg, Sjors Verlaan, Jaimie Hemsworth, Donja M. Mijnarends, Jos M. G. A. Schols, Yvette C. Luiking, Lisette C. P. G. M. de Groot

**Affiliations:** 1 Nutricia Research, Nutricia Advanced Medical Nutrition, Uppsalalaan 12, PO Box 80141, 3508TC, Utrecht, The Netherlands; 2 Department of Health Services Research, School CAPHRI, Maastricht University, Maastricht, The Netherlands; 3 Department of Family Medicine, School CAPHRI, Maastricht University, Maastricht, The Netherlands; 4 Division of Human Nutrition, Wageningen University, Wageningen, The Netherlands

**Keywords:** Micronutrients, Inadequacies, Intakes, Community-dwelling, Older adults

## Abstract

Micronutrient deficiencies and low dietary intakes among community-dwelling older adults are associated with functional decline, frailty and difficulties with independent living. As such, studies that seek to understand the types and magnitude of potential dietary inadequacies might be beneficial for guiding future interventions. We carried out a systematic review following the Preferred Reporting Items for Systematic Reviews and Meta-Analyses statement. Observational cohort and longitudinal studies presenting the habitual dietary intakes of older adults ( ≥ 65 years) were included. Sex-specific mean (and standard deviation) habitual micronutrient intakes were extracted from each article to calculate the percentage of older people who were at risk for inadequate micronutrient intakes using the estimated average requirement (EAR) cut-point method. The percentage at risk for inadequate micronutrient intakes from habitual dietary intakes was calculated for twenty micronutrients. A total of thirty-seven articles were included in the pooled systematic analysis. Of the twenty nutrients analysed, six were considered a possible public health concern: vitamin D, thiamin, riboflavin, Ca, Mg and Se. The extent to which these apparent inadequacies are relevant depends on dynamic factors, including absorption and utilisation, vitamin and mineral supplement use, dietary assessment methods and the selection of the reference value. In light of these considerations, the present review provides insight into the type and magnitude of vitamin and mineral inadequacies.

One of the most profound current shifts in demographics is the rapidly increasing population of older adults. The world population of people older than 60 years has gone from slightly more than 100 million in 1950 to more than 800 million in 2011/2012, and it is expected to exceed 2 billion by the year 2050^(^ ^1^ ^)^. Within the older population itself, there is an annual increase of 4 % in the number of people older than 80 years^(^ ^1^ ^)^. Ageing is often seen as being synonymous with frailty and disability. However, there is significant variation in age-related functional changes in older adults and, as such, widely varying dietary and nutritional needs. In the Netherlands, for example, there is a high prevalence of undernutrition among community-dwelling older adults^(^ ^2^ ^)^. Variation exists in the nutritional needs of this population, as about of the general population 11 % older than 65 years are undernourished, and this is tripled to 35 % among a population of older adults receiving home care^(^ ^2^ ^)^. The aetiology of undernutrition among older adults is complex and related to intrinsic factors, such as changes in the absorption and utilisation of nutrients and chronic disease, as well as extrinsic factors, such as poor appetite, interactions with medications, reduced enjoyment/skill in meal preparation and consumption^(^ ^3^ ^)^ and changes in the types and amounts of foods consumed^(^ ^4^ ^)^. Multiple micronutrient inadequacies among older community-dwelling adults are well described in the literature^(^ ^5^ ^,^ ^6^ ^)^. Micronutrient inadequacies appear to worsen with increasing age^(^ ^7^ ^)^, which is associated with decreased energy intakes^(^ ^5^ ^)^. There is a compound effect of micronutrient deficiencies in which an increasing number of deficient nutrients is associated with an increased incidence of frailty (hazard ratio 1·12, 95 % CI 1·03, 1·22, *P*= 0·01)^(^ ^8^ ^)^. Micronutrient deficiencies pose a considerable threat to independence and longevity, because they are related to several adverse functional outcomes^(^ ^9^ ^)^.

To our knowledge, there has not been any other systematic review of micronutrient intakes among community-dwelling older adults in developed Western countries in the literature. In light of the growing presence of this segment of the population, as well as changing and diverse nutritional needs, the present systematic review fills an important knowledge gap.

The objectives of the present systematic review were (1) to describe the habitual dietary intake of micronutrients and (2) to describe the percentage of the population at risk for inadequate intakes of micronutrients among community-dwelling older adults in Western countries.

## Methods

The present systematic review followed the reporting checklist as part of the Preferred Reporting Items for Systematic Reviews and Meta-Analyses statement^(^ ^10^ ^)^.

### Search strategy and selection of studies

The electronic databases PubMed and EMBASE were searched between the following dates: 1950 to 6 October 2011 and 1993 to 6 October 2011. The review was later updated and the same search terms were used in both databases for a search between October 2011 and 31 December 2013. The following search string was used for the searches: (‘elderly’ OR ‘geriatric’ OR ‘older adults’ OR ‘older people’ OR ‘senior’ OR ‘older person’ OR ‘aging’) AND (‘nutritional status’ OR ‘nutrient deficiency’ OR ‘nutrient deficiencies’ OR ‘nutrient deficient’ OR ‘nutrient intake’ OR ‘nutritional intake’ OR ‘food intake’ OR ‘dietary intake’ OR ‘dietary adequacy’ OR ‘nutrition assessment’ OR ‘diet records’) AND (‘population-based study’ OR ‘longitudinal study’ OR ‘epidemiologic study’ OR ‘cohort study’ OR ‘prospective study’ OR ‘cross-sectional study’ OR ‘population-based design’ OR ‘longitudinal design’ OR ‘epidemiologic design’ OR ‘cohort design’ OR ‘prospective design’ OR ‘cross-sectional design’). All possible articles were merged into one database, and duplicate records were removed. Additional articles were identified by checking the reference lists of the relevant articles, in addition to searching for national dietary/food consumption surveys. The titles and abstracts of all studies were scanned independently by two reviewers (S. t. B. and D. M. M. during the first search period and S. t. B. and J. H. during the second search period). Studies were considered eligible if they: contained nutrient intake data, were not based on a randomised controlled trial or nutrition intervention, had participants with a mean age of ≥ 65 years and had data originating from Western countries (Europe, North America, Australia or New Zealand).

Full-text articles were then assessed (by S. t. B. and J. H.) based on these selection criteria as well as the following additional criteria: if they studied community-dwelling older adults, if they had non-adjusted data^(^ ^11^ ^,^ ^12^ ^)^ and if micronutrient intake data were stated. Community-dwelling older adults were defined as those living at home, living in private households, independently living, free living or being non-institutionalised. Studies stating only the overall (men and women combined) nutrient intake data were excluded because of the separate nutrient requirements for men and women. A third reviewer (Y. C. L.) was consulted if it was unclear whether or not the article met the inclusion criteria.

### Quality assessment and data extraction

The quality of the included articles and the potential bias on the outcome was assessed based on a scale that combined the Newcastle–Ottawa quality assessment scale^(^ ^13^ ^)^ and the Cochrane coding manual for cohort studies^(^ ^14^ ^)^, using the criteria applicable for observational studies. Table 1 summarises the criteria and point assignment for the quality assessment. Summary quality scores of 0–2, 3–4 and 5 were rated as low, moderate and high, respectively. Studies were then categorised according to these ratings. Nutrient intake data from national food consumption surveys were extracted from the European Nutrition and Health Report^(^ ^15^ ^)^ and the European Food Safety Authority 2012 report^(^ ^16^ ^)^. The original articles were, however, used to assess study quality, because the reports did not contain detailed information on the quality criteria. The following study characteristics were extracted (Table 2): sample size, age range, dietary assessment method and country of origin. For each of the included studies, the mean (and standard deviation) ‘habitual’ dietary intakes of micronutrients were extracted by sex and age category. Articles were checked for reporting on potential supplement intake (yes/no) and whether supplement intake was included in their analyses (Table 2). Where data were presented as being stratified by sex and by an additional category (e.g. cognitive status), the pooled mean and standard deviation were calculated by sex group. To compare nutrient intake data with nutritional reference values, data were extracted by sex and subgroup (i.e. age category, country and year of data collection). In cases of longitudinal studies, baseline data were used, or when baseline data were not provided in the article, the follow-up measurement data were used.

**Table 1:** Overview of the study quality assessment*

Component	Criteria	Points awarded
Predefined study population (e.g. area, inclusion period)	Information provided	1
No information provided	0
Inclusion and exclusion criteria	Clearly stated	1
Not stated	0
Validated method as stated by EURRECA^(^ ^57^ ^)^ (validated FFQ, dietary history, 24 h dietary recall, diet records with ≥ 3 d or, if less, adjustment for intra-individual variability)		Method as outlined by EURRECA	2
Method as outlined by EURRECA, no statement of validation	1
Other method or information about method not provided	0
Selective reporting bias	Reported data correspond with initial sample size	1
	Reported data do not correspond with initial sample size, rationale provided	1
	Reported data do not correspond with initial sample size, no information or incomplete rationale provided	0

EURRECA, European Micronutrient Recommendations Aligned.

* Summary score: 0–2 points = low quality, 3–4 points = moderate quality, 5 points = high quality.

**Table 2:** Characteristics of the included studies, assessing nutrient intake in community-dwelling older adults

Reference	Country	Study year	Age (years)	Subjects (*n*)	Method	Supplement intake	Quality rating
Reported	Included in analysis
Adamson *et al.* ^(^ ^58^ ^)^	UK	2003–2004	≥ 85	82	FFQ	No	No	Moderate
Bates *et al.* ^(^ ^59^ ^)^	UK	2008–2010	≥ 65	224	4 d DR	Yes	Yes	Moderate
Becker *et al.* ^(^ ^15^ ^,^ ^60^ ^)^	Sweden	1997–1998	65–74	122	7 d DR	No	No	Low
Boilson *et al.* ^(^ ^61^ ^)^	Ireland	2001–2002	60–81	135	FFQ	Yes	Yes	Moderate
Castetbon *et al.* ^(^ ^15^ ^,^ ^62^ ^)^	France	2006–2007	≥ 65	349	3 × 24HR	No	No	Moderate
Decarli *et al.* ^(^ ^63^ ^)^	Switzerland	1988–1989	70–75	150	3 d DR	Yes	Unclear	Low
Elmadfa *et al.* ^(^ ^15^ ^,^ ^64^ ^)^	Austria	2007	≥ 65	349	3 d DH	Yes	No	Moderate
Elmadfa *et al.* ^(^ ^15^ ^)^	Romania	2006	≥ 65	342	Interview	Yes	No	NA
Feart *et al.* ^(^ ^65^ ^)^	France	2001–2002	≥ 75	1595	1 × 24HR	No	No	Moderate
Fidanza *et al.* ^(^ ^66^ ^)^	Italy	1981	65–69, ≥ 70	207	DH	No	No	Moderate
Finch *et al.* ^(^ ^67^ ^)^	UK	1994–1995	≥ 85	266	4 d weighed DR	Yes	Unclear	Moderate
Gibson^(^ ^68^ ^)^	UK	1994–1995	≥ 65	806	4 d weighed DR	Yes	Unclear	Moderate
Griep *et al.* ^(^ ^69^ ^)^	Belgium	NA	60–75	91	7 d DR	No	No	Moderate
Health Canada *et al.* ^(^ ^24^ ^)^	Canada	2004	≥ 70	4130	1 × 24HR	Yes	No	High
Horwath *et al.* ^(^ ^70^ ^)^	New Zealand	1988	≥ 70	712	FFQ	Yes	Unclear	High
Hulshof *et al.* ^(^ ^15^ ^,^ ^71^ ^)^	Netherlands	1997–1998	≥ 65	421	2 d DR	Yes	No	Moderate
Johansson *et al.* ^(^ ^15^ ^,^ ^72^ ^)^	Norway	1997	≥ 65	342	FFQ	Yes	Yes	Moderate
Konstantinova *et al.* ^(^ ^73^ ^)^	Norway	1997–1999	71–74	2855	FFQ	Yes	Yes	Moderate
Lopes *et al.* ^(^ ^15^ ^,^ ^74^ ^)^	Portugal	NA	≥ 65	585	FFQ	Yes	No	Moderate
Luhrmann *et al.* ^(^ ^75^ ^)^	Germany	1994	60–85	308	3 d DR	No	No	High
Max Rubner-Institut^(^ ^15^ ^,^ ^76^ ^)^	Germany	2005–2007	≥ 65	3031	DH	Yes	Unclear	Moderate
Milman *et al.* ^(^ ^77^ ^)^	Denmark	1994–1995	80	240	3 d DR	Yes	Yes	High
Mowe *et al.* ^(^ ^78^ ^)^	Norway	1989	70–91	95	DH	Yes	Unclear	Moderate
Nelson *et al.* ^(^ ^79^ ^)^	USA	1995	≥ 65	3634	FFQ	Yes	Yes	High
Nicolas *et al.* ^(^ ^80^ ^)^	France	1993, 1995, 1997	60–94	262	3 d DR	No	No	Moderate
Ocke *et al.* ^(^ ^49^ ^)^	Netherlands	2010–2012	≥ 70	739	2 × 24HR	Yes	Yes*	High
Ortega *et al*.^(^ ^81^ ^)^	France	1995	≥ 70	260	7 d weighed DR	Yes	No	High
Pedersen *et al*.^(^ ^15^ ^,^ ^82^ ^)^	Denmark	2003–2008	≥ 65	438	7 d DR	No	No	Moderate
Pietinen *et al.* ^(^ ^15^ ^,^ ^83^ ^)^	Finland	2007	≥ 65	463	48HR	Yes	Yes	Low
Posner *et al.* ^(^ ^84^ ^)^	USA	NA	70–79	1154	24HR	No	No	Low
Rothenberg *et al*.^(^ ^85^ ^)^	Sweden	1971, 1981, 1993	70	360	DH	No	No	Moderate
Serra Majem *et al.* ^(^ ^15^ ^,^ ^86^ ^)^	Spain	2002–2003	≥ 65	342	2 × 24HR	No	No	Low
Sette *et al.* ^(^ ^15^ ^,^ ^87^ ^)^	Italy	2005–2006	≥ 65	518	3 d DR	Yes	No	Moderate
Szponar *et al.* ^(^ ^15^ ^,^ ^88^ ^)^	Poland	2000	≥ 65	453	24HR	No	No	Low
Toffanello *et al*.^(^ ^89^ ^)^	Italy	1989–1999	70–75	78	DH	No	No	Moderate
USDA *et al.* ^(^ ^90^ ^)^	USA	2005–2006	≥ 70	997	2 × 24HR	No	No	High
Zoltick *et al.* ^(^ ^91^ ^)^	USA	1988–1989	67–93	807	FFQ	Yes	Yes	High

DR, dietary record; 24HR, 24 h dietary recall; DH, dietary history; NA, not applicable because data was not available; 48HR, 48 h dietary recall; USDA, US Department of Agriculture.

* Data were published with and without supplement intake included; habitual intake (without supplement intake) was used in the analysis for the present systematic review.

### Data analysis

All analyses were done in IBM SPSS Statistics version 19.0 (2010, IBM Company). Graphics were done in GraphPad Prism version 6.00 for Windows (GraphPad Software).

Pooled means and standard deviations were calculated by sex for each nutrient. We performed a sensitivity analysis using a one-way ANOVA comparing the mean nutrient intakes in each study-quality subgroup with an *ad hoc* least significant difference test to assess between-group differences. Significant differences in micronutrient intakes by quality subgroup were defined as *P*< 0·05.

Micronutrient estimated average requirements (EAR) from the Nordic Nutrition Recommendations^(^ ^17^ ^)^ were used for most nutrients. The Institute of Medicine's EAR was used for Mg^(^ ^18^ ^)^, because it was not provided in the Nordic Nutrition Recommendation. In addition, the updated Institute of Medicine's EAR for vitamin D and Ca^(^ ^19^ ^)^ were used. Adequate intake values were used for K and Na, because there are not yet EAR for these nutrients for the older age group^(^ ^20^ ^)^. Sex-specific and age-specific (older than 60 years) recommendations were used if stated. The EAR cut-point method^(^ ^21^ ^)^ was used to calculate the prevalence of inadequate intakes for each nutrient. This method assumes normal distribution of both the population intakes and the recommendation. Because the EAR is a recommendation that meets the needs of at least 50 % of the population, the mean and standard deviation of the intakes (when normally distributed) can be used to calculate the percentage of the population that are falling below the recommendation and as such are at risk for inadequacy. Nutrients were considered to be a potential concern when the prevalence of inadequate intakes was equal to or above 30 % of the population for both men and women. For K and Na, the mean intake was compared with the adequate intake in order to make a qualitative comparison. If the intake was above the adequate intake, a low prevalence of inadequacy was assumed. If the intake was below the adequate intake, the inadequacy could not be determined.

## Results

**Fig. 1:**
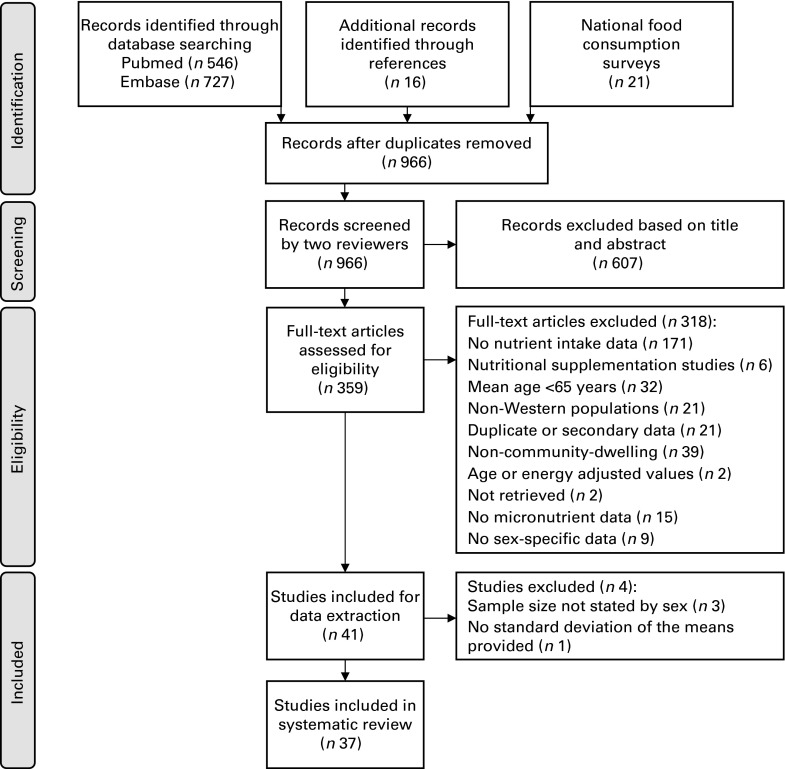
Systematic reviews and meta-analyses (Preferred Reporting Items for Systematic Reviews and Meta-Analyses) flowchart of article selection and inclusion.

A total of 966 articles were identified as potentially relevant from the two searches (Fig. 1). This resulted in thirty-seven separate articles from more than 28 000 (57 % female) community-dwelling older adults in twenty different Western countries (Table 2). There was a range in individual study quality – twenty-one of the thirty-seven studies were of moderate quality, six were of low quality, nine were of high quality, and one article's quality could not be assessed due to insufficient information (Table 2; see online supplementary Table S1 for full quality assessment). The results of the sensitivity analysis showed no significant differences (*P*>0·05) between mean nutrient intakes in each of the three quality subgroups. The cut-point analysis was, therefore, derived from the means and standard deviations of the full sample (see online supplementary Tables S2–S5 for the dietary intake data from each study).

### Habitual vitamin intakes and percentage at risk

**Fig. 2:**
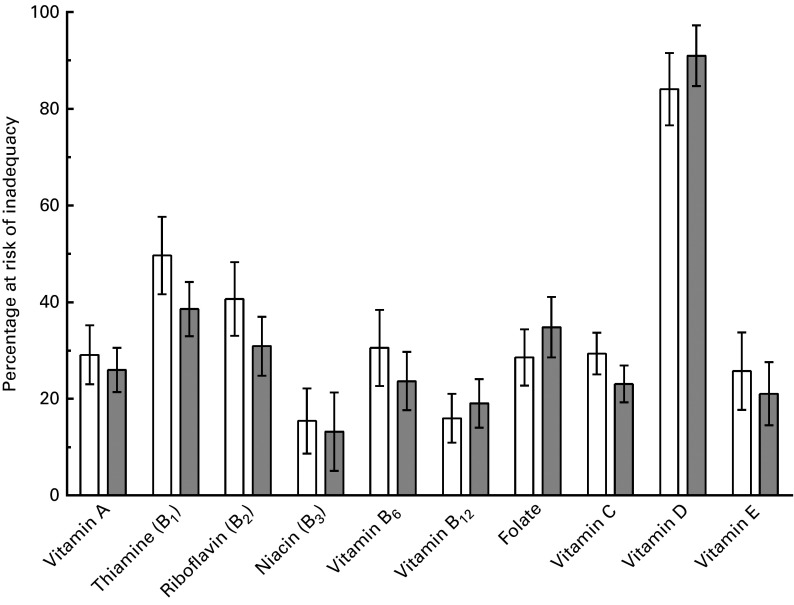
Mean (95 % CI) percentage of men (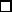) and women (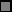) at risk for inadequate intake of vitamins.

The mean dietary intakes of ten vitamins (vitamin A, thiamin (B_1_), riboflavin (B_2_), niacin (B_3_), vitamins B_6_ and B_12_, folate and vitamins C, D and E) by both men and women are summarised in Table 3. The percentage of the population at risk for inadequate intakes of vitamins from food alone was greater than 30 % for both men and women for three of the ten analysed vitamins: thiamin, riboflavin and vitamin D (Fig. 2). Half of the male population was at risk for inadequate intake of thiamin as compared to the female population, where one-third (39 %) was at risk for an inadequacy. Fewer men and women were at risk for riboflavin inadequacy, with 41 and 31 % for men and women, respectively, having inadequate intakes. Most men and women were at risk for inadequate dietary intakes of vitamin D (84 and 91 % for men and women, respectively). Vitamins that showed lower rates of inadequacy but that could also be a potential dietary concern include: vitamin A (29 and 26 % for men and women, respectively), vitamin B_6_ (31 and 24 %), folate (29 and 35 %), vitamin C (29 and 23 %) and vitamin E (26 and 21 %).

**Table 3:** Daily vitamin intake and percentage of inadequate intakes among older adults (Mean values and standard deviations; percentages and 95 % confidence intervals)

Nutrient	Sex	Studies (*n*)	Pooled (*n*)	Unit	EAR	Mean	sd	Percentage below EAR*	95 % CI
Vitamin A	M	30	7985	μg RE/d	600	1273	489	29	23, 35
	W	30	10 839		500	1133	472	26	21, 31
Thiamin (B_1_)	M	31	9351	mg/d	1·2	1·3	0·3	50	42, 58
	W	31	12 380		0·9	1·1	0·3	39	33, 44
Riboflavin (B_2_)	M	30	9284	mg/d	1·4	1·7	0·4	41	33, 48
	W	30	12 266		1·1	1·5	0·4	31	25, 36
Niacin (B_3_)	M	16	5408	mg/d	15	27	7	15	9, 22
	W	16	7013		12	23	6	13	5, 21
Vitamin B_6_	M	22	8140	mg/d	1·3	1·8	0·4	31	23, 38
	W	22	10 837		1	1·5	0·4	24	18, 30
Vitamin B_12_	M	19	7660	μg/d	1·4	6·4	1·5	16	11, 21
	W	19	10 352		1·4	5·1	1·3	19	14, 24
Folate	M	22	9876	μg/d	200	278	61	29	23, 34
	W	22	12 917		200	253	53	35	29, 41
Vitamin C	M	35	8779	mg/d	60	99	25	29	25, 34
	W	35	11 694		50	103	29	23	19, 27
Vitamin D	M	24	7873	μg/d	10	5·4	2·7	84	77, 92
	W	24	10 291		10	4·5	2·4	91	85, 97
Vitamin E	M	17	4973	α-TE/d	6	9·6	3·0	26	18, 34
	W	17	6150		5	8·7	2·6	21	15, 28

EAR, estimated average requirement; M, men; RE, retinol equivalent; W, women; TE, tocopherol equivalent.

* Mean percentage of inadequate intakes, calculated with the EAR cut-point method.

### Habitual mineral intakes and percentage at risk

The mean dietary intakes of ten minerals (Ca, Cu, I, Fe, Mg, P, K, Se, Na and Zn) by both men and women are summarised in Table 4.

**Table 4:** Daily mineral intake and percentage of inadequate intakes among older adults (Mean values and standard deviations; percentages and 95 % confidence intervals)

Nutrient	Sex	Studies (*n*)	Pooled (*n*)	Unit	EAR	Mean	sd	Percentage below EAR*	95 % CI
Ca	M	36	9173	mg/d	1000	864	159	65	59, 71
	W	36	12 378		1000	795	130	73	68, 78
Cu	M	7	1690	mg/d	0·7	1·4	0·2	14	7, 22
	W	7	1956		0·7	1·2	0·3	18	10, 25
I	M	8	1439	μg/d	100	181	57	20	10, 30
	W	8	1710		100	159	59	26	12, 41
Fe	M	31	8195	mg/d	7	14	3	11	8, 15
	W	31	10 911		6	11	3	12	9, 15
Mg	M	20	7042	mg/d	350	296	35	73	66, 80
	W	20	9437		265	294	51	41	32, 50
P	M	13	4532	mg/d	450	1326	156	11	0, 25
	W	13	6397		450	1142	133	12	0, 27
K	M	17	6581	g/d	4·7†	3·2	0·5	NA‡
	W	17	8778		4·7†	2·8	0·4	NA‡
Se	M	10	2227	μg/d	35	53	19	30	21, 38
	W	10	2582		30	43	14	30	22, 37
Na	M	15	5467	g/d	1·3†	3·1	0·6	NA, low prevalence of inadequacy§
	W	15	6978		2·4†	2·5	0·5	NA, low prevalence of inadequacy§
Zn	M	18	6510	mg/d	6	10	2	12	8, 17
	W	18	8786		5	9	1	12	6, 17

EAR, estimated average requirement; M, men; W, women; NA, not applicable.

* Mean percentage of inadequate intakes, calculated with the EAR cut-point method.

† Adequate intake, thus unable to apply EAR cut-point method.

‡ Mean intake was below the adequate intake; no conclusion can be made about inadequacy.

§ Mean intake is above adequate intake; a low prevalence of inadequacy is assumed.

**Fig. 3:**
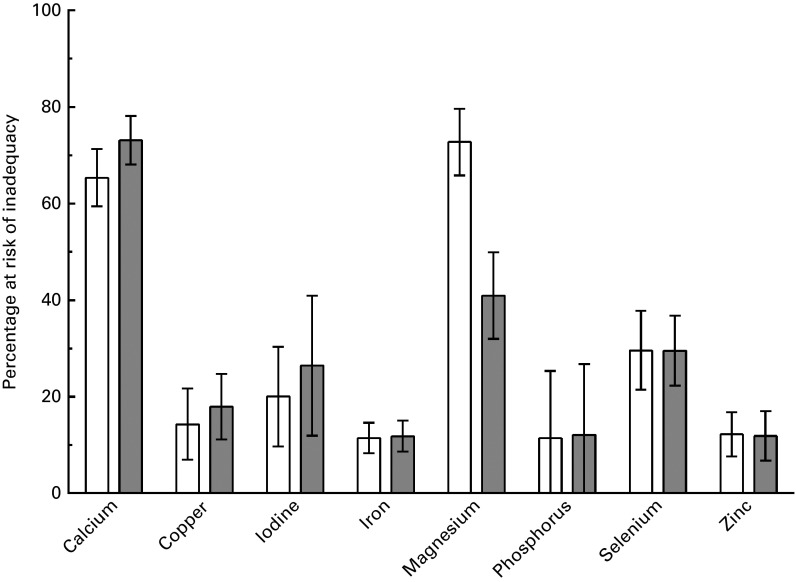
Mean (95 % CI) percentage of men (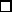) and women (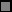) at risk for inadequate intake of minerals.

The percentage of the population at risk for inadequate dietary intakes of minerals from food alone was equal to or greater than 30 % for both men and women for three of the analysed minerals: Ca, Mg and Se. Fig. 3 shows the percentage at risk of inadequacy; two nutrients show a clear ‘higher’ risk for inadequacy than the other nutrients. Nearly two-thirds (65 %) of the population of men had inadequate intakes of Ca, and three-quarters (73 %) of the population of women were at risk for inadequacy for Ca. Almost three-quarters (73 %) of the population of men and nearly half (41 %) of the population of women were at risk for inadequate intakes of Mg. For both men and women, 30 % were at risk of Se inadequacy. Finally, iodine showed a potential risk for inadequate intakes, with 20 % of men and 26 % of women being at risk.

## Discussion

The present systematic review identified six nutrients of potential concern as a result of a high prevalence of inadequacies – thiamin, riboflavin, vitamin D, Ca, Mg and Se. The results from the present systematic review support previous reports of low micronutrient adequacy in older adult diets in Europe^(^ ^22^ ^,^ ^23^ ^)^. Potential vitamin D, Ca, Mg and Se inadequacies were also identified in younger adult populations (aged 19 years and older)^(^ ^24^ ^,^ ^25^ ^)^. This suggests that these inadequacies might not be confined to the older adult population.

Whether these low micronutrient intakes are of true public health concern and the other nutrients are not of public health concern depends on several factors. In older adults, the picture of nutritional status is not complete without also considering nutrient absorption, including sun exposure in the case of vitamin D, and utilisation as assessed by biochemical status, micronutrient supplementation use and potential differences in the nutrient requirements/recommendations upon which the percentage at risk calculation is based. Therefore, the results of the present systematic review are to be interpreted in light of these dynamic factors.

### Nutrient absorption, utilisation and biochemical status

#### Vitamin D

A high proportion of the population has low intakes of vitamin D, because dietary sources are rare and are limited to fatty fish and, in some cases, dairy products^(^ ^26^ ^)^. Most of the vitamin D we use is delivered through skin synthesis and/or dietary supplement intake^(^ ^27^ ^)^. In addition, fortified foods can contribute to vitamin D intake. Nevertheless, serum concentrations of 25-hydroxyvitamin D (25(OH)D) remain deficient ( < 50 nmol/l) in 40–100 % of senior populations globally^(^ ^26^ ^,^ ^28^ ^)^. Vitamin D deficiencies have been related to fractures, falls and low physical performance and potentially also to age-related cognitive decline^(^ ^26^ ^)^. Among the thirty-eight studies we reviewed, three published 25(OH)D levels for the same participants for the same period of dietary intake. The mean 25(OH)D level in these studies was 56·2 (sd 14·0) nmol/l among men and 51·7 (sd 9·6) nmol/l among women. Assuming normal distribution, this suggests that approximately half of the population in these studies is deficient in vitamin D, which agrees with the estimated range of deficiencies among older adults^(^ ^26^ ^)^. A study of community-dwelling older adults in Canada showed that higher intakes of vitamin D (both dietary and supplementary) result in a higher adequacy of 25(OH)D levels^(^ ^29^ ^)^. The sample was stratified by a combined dietary and supplementary intake of 20 μg/d, where 34 % of the sample was below and 66 % of the sample was above this intake level. Of the sample that had intake below this level, 35 % had deficient 25(OH)D levels of < 50 nmol/l, and of those who had intake above this level, only 2 % had deficient 25(OH)D levels. Of the adequate consumers, 73 % had sufficient 25(OH)D levels ( ≥ 75 nmol/l). Therefore, the habitual intake of vitamin D observed in the present study is alarming considering the worldwide prevalence of vitamin D deficiencies. Higher dietary and supplementary intakes of vitamin D result in the reversal of vitamin D deficiencies and an increase in serum 25(OH)D concentrations among community-dwelling older adults^(^ ^30^ ^)^. As such, this is a worthwhile intervention for preventing and reversing vitamin D deficiencies.

#### Calcium and magnesium

Considering the high prevalence of dietary inadequacy of Ca and Mg, measures of actual status would be useful to interpret whether these nutrients pose true health concerns at a population level. However, biomarkers for Ca and Mg are generally thought to be problematic because they have no specific useful measurement technique^(^ ^31^ ^)^. The functional outcome of Ca intake is often bone density, where higher intakes of Ca (>500 mg/d) plus vitamin D_3_ are associated with a higher bone density^(^ ^30^ ^)^ and thus a decreased risk of fractures. However, Ca absorption is dependent on vitamin D intake, because vitamin D facilitates the intestinal absorption of Ca^(^ ^32^ ^)^. Mg is also thought to be involved in the development of healthy bones, and it could play a role in muscle mass development^(^ ^33^ ^)^ and muscle performance in older adults^(^ ^34^ ^)^.

#### B vitamins

The prevalence of inadequate intakes of thiamin (B_1_) and riboflavin (B_2_) were of concern for both men and women. Although subclinical deficiencies of these nutrients have been reported and have been linked with cognitive outcomes^(^ ^35^ ^)^, there does not appear to be a large public health concern about this level of thiamin and riboflavin dietary intakes.

The prevalence of inadequate vitamin B_6_ intake among the present pooled population was on the threshold of being a concern, because 31 % of men and 24 % of women were at risk of having inadequate intakes. Although it is an essential nutrient, vitamin B_6_ is not thought to be a typical nutrient of concern because it is fairly ubiquitous in Western diets^(^ ^36^ ^)^. However, vitamin B_12_, which is apparently adequate through habitual intakes, is frequently deficient in the blood values of older adults^(^ ^37^ ^)^. Malabsorption of vitamin B_12_ is the primary cause of the low vitamin B_12_ status among older adults, because atrophy of the gastric folds impairs gastric acid production, which reduces the activity of intrinsic factors that are essential for the absorption of vitamin B_12_^(^ ^37^ ^)^. Even high intakes of vitamin B_12_ from dietary and supplementary sources have a plateau effect in increasing serum concentrations because there is less efficient absorption with higher intakes^(^ ^38^ ^)^. Low levels of vitamin B_12_ have been linked with an increased risk of fractures^(^ ^39^ ^)^, and less robust evidence exists for a relationship between vitamin B_12_ status and cognitive function^(^ ^40^ ^)^. Homocysteine, an α-amino acid that becomes elevated in plasma when vitamin B_6_, vitamin B_12_ or folate levels are suboptimal, is raised in 30–50 % of populations of adults aged 60 years or older (reviewed in van Wijngaarden *et al.* ^(^ ^39^ ^)^). Elevated homocysteine levels are significantly associated with bone fracture risk^(^ ^39^ ^)^, are an independent risk factor in CVD^(^ ^41^ ^,^ ^42^ ^)^ and are implicated in the reduced physical function of older adults^(^ ^43^ ^)^. There is evidence that vitamin B_12_, folate and perhaps vitamin B_6_ play a role in reducing homocysteine levels^(^ ^44^ ^)^.

#### Antioxidants (selenium and vitamins A, C and E)

In the present pooled population, a high proportion of inadequate intakes of Se was observed in both men and women. Clinical deficiencies are rare, but higher serum Se concentrations have been associated with protective effects against cancer and anaemia^(^ ^45^ ^,^ ^46^ ^)^. The hypothesised mechanism for anaemia protection is through Se's antioxidant activity, which prevents erythrocyte oxidation and damage^(^ ^45^ ^)^. However, the link between Se intake and serum Se concentrations, especially among elderly populations, is not well understood^(^ ^45^ ^)^. One of the pathways that leads to frailty and disability among older adults is oxidative stress^(^ ^8^ ^)^. Serum carotenoids and serum Se were both significantly negatively related to frailty in an observational study in elderly women in the USA^(^ ^45^ ^)^. Serum α-tocopherol also showed a trend between low serum levels and frailty (*P*= 0·06). This suggests, at least among the present group of women, that antioxidants play a strong role in the development of frailty and disability, independent of other background factors, such as smoking, educational attainment and chronic disease. We observed a borderline (20–30 %) high prevalence of vitamin A, C and E dietary inadequacies. Although serum markers are questioned for their reliability concerning dietary intake^(^ ^47^ ^)^, serum markers of antioxidants are linked with frailty and disability. The present results suggest that this pooled population of older adults could have important dietary shortages of antioxidants.

### Vitamin and mineral supplement intake and adequacy of intakes

Habitual dietary intakes of micronutrients among older adults are of course only part of the total picture of micronutrient intake, as the proportion of seniors who take vitamin and mineral supplements is steadily on the rise^(^ ^48^ ^)^. In the Netherlands, during the period between 2010 and 2012, approximately 26 % of women and 18 % of men took vitamin D supplements of at least 10 μg throughout the year. Slightly higher percentages took the same amount of vitamin D daily during the winter months only^(^ ^49^ ^)^. A German study in 2009 among older adults showed a high proportion of the population consuming vitamin and mineral supplements regularly – 34 % of men and 54 % of women^(^ ^50^ ^)^. Regular consumption of individual nutrients such as vitamin D was much lower, with 7 % of men and 19 % of women taking between 7·4 and 10 μg/d. About 14 % of men and 22 % of women were regular consumers of Mg supplements, but the number that met or exceeded the recommendation remained low, at 16 % of men and 18 % of women. According to the Canadian Community Health Survey (micronutrient intakes from foods included in the present report), 45 % of male and 60 % of female older adults in Canada reported consuming supplements during the period of the dietary data collection^(^ ^24^ ^)^. Another study in a representative population of older Canadian adults (>51 years) showed a high prevalence of supplement use (40 %)^(^ ^51^ ^)^. For micronutrients that had observed high risks of inadequacies from dietary intake alone (Mg, Zn, Ca, vitamin A, vitamin C and vitamin D), dietary supplements appeared to close the nutrient gap, with the exception of vitamin D, Mg and Ca, where between 12 and 38 % of the adults remained below the EAR^(^ ^51^ ^)^. This is consistent with another study among older adults in Austria, where older adults who consumed dietary supplements were compared with older adults who did not consume dietary supplements. Vitamin D deficiency (25(OH)D < 50 nmol/l) still existed in 88 % of the total population, whereas 18 % of the supplemented group had adequate status *v*. 4 % in the control group^(^ ^48^ ^)^.

### Nutrient recommendations and dietary assessment methods influencing the interpretations of micronutrient intake adequacy

One of the largest problems related to dietary assessment and inter-group comparisons is the lack of harmonisation in nutrient recommendations^(^ ^23^ ^,^ ^52^ ^)^. For example, there are twenty-two different recommendations for vitamin D cited for adults aged 70 years or older in Europe^(^ ^53^ ^)^. These range from 2·5 to 15 μg/d, with a median or 7·5 μg/d for men and 10 μg/d for women. The most frequently used value was 10 μg/d (which was the EAR used for both men and women in the present review). Therefore, the percentage of the population at risk for inadequacy is sensitive to the recommendation that is selected. Comparison to a recommendation from another expert committee could therefore influence the present conclusions.

A practical example of the effects of recommendations on calculating the percentage at risk for inadequacy occurred in the present dataset. There was a large sex difference between the percentages at risk for inadequate intakes observed with Mg. Although the Mg intakes were similar (with mean intakes of 296 and 294 mg/d for men and women, respectively), the percentage at risk of inadequate intake was substantially different (73 % for men and 41 % for women) because the EAR are 350 mg/d for men and 265 mg/d for women. Although the Institute of Medicine Mg recommendations contain age- and sex-specific EAR, the scientific evidence supporting these recommendations is limited. Mg balance studies were used, and studies were absent for several age categories and for women in particular. Differences in total energy consumption, and therefore in Mg consumption, between men and women might have influenced the recommendations. Although the most recent age-specific EAR were chosen for the present comparison, the differences between the scientific substantiation of the nutritional recommendations should be considered. In general, there is a need for high-quality markers of nutritional status and for studies performed in (community-dwelling) older adults. Because the recommendations used were published in 1997–2011, more recent insights (e.g. those based on intervention studies on functional outcomes) may affect the present conclusion.

In addition to recommendations, dietary assessment methods also influence the calculation of the population at risk for inadequate intake^(^ ^53^ ^,^ ^54^ ^)^. Dietary surveys sometimes rely on memory recalls from older adults, and it is unknown to what extent memory impairments and cognitive functioning influence the reliability of the data^(^ ^3^ ^)^. As stated by Ribas-Barba *et al.* ^(^ ^53^ ^)^, there is currently no perfect dietary assessment method for measuring usual intake. Each measurement has its advantages and disadvantages, and each has its own appropriateness regarding the unique needs of the population and the objective. Moreover, the threshold we selected ( ≥ 30 % at risk for inadequacy) to define nutrients of potential concern included a buffer to account for dietary assessment error^(^ ^55^ ^)^.

Another factor that influences inter-group comparison is the different food composition tables that are used to calculate nutrient intake. The quality and content of food composition tables often differ by country, and this may have introduced variation among the studies included in the present analysis. The time span of the included studies should also be considered, because dietary habits and food compositions change over time.

### Strengths and limitations

The present study has a few limitations which should be mentioned. The main limitation is that it represents a small part of a larger clinical picture of intake, absorption, supplement/medication use and functional impairments or outcomes. Although examining all aspects simultaneously in such a large population is not possible, it is difficult to interpret information about the dietary inadequacies of older adults without also considering the other factors. We have attempted to carefully examine each of these dynamic areas and to position our conclusions within this theoretical context. However, it is important to mention that monitoring the status of micronutrients is important in senior populations, even though intake and status are not always well correlated. One example is 25(OH)D status, which is determined not only by nutritional intake but also by sun exposure. Another example is Ca status, for which no accurate marker is currently available.

Many studies did not report whether the intake came from food alone or whether supplement intakes were included in the estimations. In addition, studies that did report supplement intake often only stated the percentage of supplement users and not the types or amounts of supplemented nutrients. This illustrates that there is a need for assessing and better reporting supplement intake in the older adult population. As the proportion of older adults who consume supplements increases^(^ ^56^ ^)^, this is becoming an important methodological concern, because it affects our insight into the true extent of micronutrient inadequacies among this population. In the present data analysis, the studies that included supplement intake did not show a consistently low prevalence of nutrient inadequacies. Although information on supplement intake was limited, we do not expect that the studies that included supplement intake strongly influenced our conclusions. Food fortification might also have affected our conclusions, because we did not have insight into the food composition data. This additional source should be considered when interpreting the results for a specific country, as foods are fortified (e.g. vitamin D in dairy, iodine in discretionary salt) in some Western countries. In addition, several countries have supplementation advice (e.g. vitamin D).

Safety levels of the micronutrient intakes were not assessed in the present systematic review. However, they may be of concern for certain nutrients, such as Na. In the present pooled population, Na intake was 3·1 (sd 0·6) g/d in men and 2·5 (sd 0·5) g/d in women, which exceed the recommended upper limits of 2·3 g/d^(^ ^20^ ^)^.

The choice to include only Western populations was made in order to describe potential inadequacies in the patterns of populations that are most homogeneous. However, this choice could have excluded relevant populations, which may have affected the external validity of our findings. For example, including Japan and Brazil might attenuate or exaggerate apparent nutrients of concern given the wide diversity of traditional dietary patterns. As such, the presented results may provide a proxy for existing dietary inadequacies; however, they may not be representative of global populations of community-dwelling older adults.

We have assumed normality for the present analysis, but the distribution might have been tailed for some nutrients. As a consequence, the inadequacies for these nutrients might have been over- or underestimated.

Nevertheless, there are also strengths to the present review. The main strength is that it gives a robust overview with a large pooled sample size of dietary intakes of vitamins and minerals in Western countries. Thus, this makes the results in the present study more generalisable to Western populations as compared to those in cross-country comparisons. We used a systematic approach to evaluate the quality and risk of bias in each study, which allowed us to perform a robust sensitivity analysis between quality groups. This rigorous method allowed us to present the pooled results with confidence.

### Conclusion

In the present systematic review, we identified six nutrients which may be consumed at inadequate amounts at a population level: vitamin D, thiamin, riboflavin, Ca, Mg and Se. Although several other factors are known to influence total micronutrient intakes and, ultimately, nutrient status, the present review provides an important and robust snapshot of the types and magnitude of nutrient intake concerns among Western community-dwelling older adults.

## Supplementary material

For supplementary material accompanying this paper visit http://dx.doi.org/10.1017/S0007114515000203. click here to view supplementary material
