# Supplementary material for: Micronutrient intakes and potential inadequacies of community-dwelling older adults: a systematic review
Source: Br J Nutr. 2015 Mar 30;113(8):1195–206. doi: 10.1017/S0007114515000203 (PMC4531469; doi:10.1017/S0007114515000203)
Supplement: Supplementary file 1 [file S0007114515000203sup001.pdf]

Supplementary material

Micronutrient intakes and potential inadequacies of community-dwelling older adults. a systematic review

Sovianne ter Borg, Sjors Verlaan, Jaimie Hemsworth, Donja M. Mijnarends, Jos M.G.A. Schols, Yvette C. Luiking, Lisette C.P.G.M. de Groot

Table S1 Quality assessment based on the Newcastle-Ottawa quality assessment scale and the Cochrane coding manual for cohort studies. **2**

Table S2 Individual study data and % of the population at risk for inadequacy, vitamin intakes among older men. **3**

Table S3 Individual study data and % of the population at risk for inadequacy, mineral intakes among older men. **4**

Table S4 Individual study data and % of the population at risk for inadequacy, vitamin intakes among older women. **5**

Table S5 Individual study data and % of the population at risk for inadequacy, mineral intakes among older women. **6**

Table S1 Quality assessment based on the Newcastle-Ottawa quality assessment scale[1] and the Cochrane coding manual for cohort studies[2].

	Selection bias		Outcome bias		
Reference	Predefined population*	In/Exclusion criteria†	Validated method‡	Transparent reporting§	Quality
Adamson *et al.*[3]	1	0	2	1	moderate
Bates *et al.*[4]	1	1	1	0	moderate
Becker *et al.*[5]	1	0	1	0	low
Boilson *et al.*[6]	1	0	2	1	moderate
Castetbon *et al.*[7]	1	1	1	0	moderate
Decarli *et al.*[8]	1	1	0	0	low
Elmadfa *et al.*[9]	1	1	1	0	moderate
Elmadfa *et al.*[10]	NA	NA	NA	NA	NA
Feart *et al.*[11]	1	1	1	1	moderate
Fidanza *et al.*[12]	1	0	1	1	moderate
Finch *et al.*[13]	1	1	1	0	moderate
Gibson[14]	1	0	1	1	moderate
Griep *et al.*[15]	0	1	2	1	moderate
Health Canada *et al.*[16]	1	1	2	1	high
Horwath *et al.*[17]	1	1	2	1	high
Hulshof *et al.*[18]	1	1	1	0	moderate
Johansson *et al.*[19]	1	0	2	0	moderate
Konstantinova *et al.*[20]	1	0	2	1	moderate
Lopes *et al.*[21]	1	1	2	0	moderate
Luhrmann *et al.*[22, 23]	1	1	2	1	high
Max Rubner-Institut[24]	1	1	1	0	moderate
Milman *et al.*[25]	1	1	2	1	high
Mowe *et al.*[26]	0	1	1	1	moderate
Nelson *et al.*[27]	1	1	2	1	high
Nicolas *et al.*[28]	0	1	1	1	moderate
Ocke *et al.* [29]	1	1	2	1	high
Ortega *et al.*[30]	1	1	2	1	high
Pedersen *et al.*[31]	1	1	1	0	moderate
Pietinen *et al.*[32]	1	0	1	0	low
Posner *et al.*[33]	1	1	0	0	low
Rothenberg *et al.*[34]	1	1	1	1	moderate
Serra Majem *et al.*[35]	1	0	1	0	low
Sette *et al.*[36]	1	1	1	0	moderate
Szponar *et al.*[37]	1	0	0	0	low
Toffanello *et al.*[38]	0	0	2	1	moderate
USDA *et al.*[39]	1	1	2	1	high
Zoltick *et al.*[40]	1	1	2	1	high

NA, not applicable: the quality could not be assessed as study design details could not be retrieved; USDA, U.S. Department of Agriculture.

* Recruitment place and year were clearly stated; † In- and exclusion criteria during sample selection were clearly stated; ‡ Method was one of the following: validated FFQ, dietary history, 24h recall, dietary records of at least 3d or when less than 3d adjusted for intra-individual variability; § All older persons included in the study were accounted for or reasons for exclusion from data analysis were disclosed. Items *, †, ‡ were scored on absence (0 points) or presence (1 point) of the quality item. Item § was scored on absence (0 points), presence of appropriate method (1 point) or presence of validated appropriate method (2 points).

Table S2 Individual study data and % of the population at risk for inadequacy, vitamin intakes among older men.

		Vitamin A (µg-RE/d)			Thiamine (mg/d)			Riboflavin (mg/d)			Niacin (mg/d)			Vitamin B_6 (mg/d)			Vitamin B_{12} (µg/d)			Folate (µg/d)			Vitamin C (mg/d)			Vitamin D (µg/d)			Vitamin E (α-TE/d)		
Reference	Country	Mean	SD	%	Mean	SD	%	Mean	SD	%	Mean	SD	%	Mean	SD	%	Mean	SD	%	Mean	SD	%	Mean	SD	%	Mean	SD	%	Mean	SD	%
Adamson *et al.*[3]	UK																						121	48	10						
Bates *et al.* [4]	UK	1707	1751	26	1.9	2.2	38	2.2	2.3	37	39.0	13.9	4	2.8	2.4	27	8.1	5.4	11	315	129	19	110	105	32	5.0	4.0	89			
Becker *et al.* [5]	Sweden	1500	700	10	1.5	0.4	23	1.9	0.6	20	37.9	11.7	3	2.3	0.7	8	8.0	3.9	5	240	75	30	93	50	25	7.1	2.6	87	7.6	2.7	28
Boilson *et al.* [6]	Ireland																5.7	4.4	16	384	117	6	135	80	17	4.2	2.5	99			
Castetbon *et al.* [7]	France																														
Decarli *et al.* [8]	Switzerland	1072	424	13	0.9	0.3	84	1.5	0.5	39				1.1	0.3	74							116	51	14						
Elmadfa *et al.* [9]	Austria	1200	1600	35	1.0	0.3	75	1.2	0.4	69	25.0	8.2	11	1.2	0.4	60	4.7	2.3	8	166	68	69	102	59	24	2.9	4.5	94	13.0	4.2	5
Elmadfa *et al.* [10]	Romania	2200	900	4	1.6	0.7	28	3.2	1.7	14													77	39	33						
Feart *et al.* [11]	France	690	1784	48	1.1	0.5	55	1.7	0.7	32				1.6	0.6	28	6.0	9.5	31	302	131	22	85	64	35	1.9	2.6	100	7.1	4.9	41
Fidanza *et al.*. [12] 65-69.9y	Italy	671	704	46	0.9	0.3	83	0.9	0.3	93													55	27	57						
Fidanza *et al.* [12] 70+y	Italy	679	755	46	0.9	0.4	81	1.0	0.4	86													62	30	47						
Finch *et al.* [13]	UK																						64	103	49						
Gibson [14]	UK							1.8	0.0	50										264	83	22	65	41	45	4.0	2.1	100			
Griep *et al.* [15]	Belgium	700	330	38	1.1	0.2	70	1.0	0.2	97	16.2	4.2	39	1.5									68	42	42						
Health Canada *et al.* [16]	Canada	790	1910	46	1.7	1.6	38	1.9	1.9	41	36.4	31.2	25	1.8	1.6	37	4.4	11.7	40	425	429	30	111	156	37	6.3	15.6	59			
Horwath *et al.*[17]	New-Zealand	1531	643	7	1.2	0.4	48	1.7	0.6	29	29.7	8.0	3	1.3	0.4	48	4.9	2.5	9	233	69	32	109	68	24	2.9	1.5	100			
Hulshof *et al.* [18]	Netherlands	1100	800	27	1.4	0.7	39	1.6	0.5	34				1.8	0.5	16				204	78	48	88	56	31	4.8	2.9	96	13.7	6.5	12
Johansson *et al.*[19]	Norway	2500	1400	9	1.8	1.1	29	2.1	1.2	28	21.6	13.7	31										139	81	16	15.0	11.4	33			
Konstantinova *et al.* [20]	Norway				1.6	0.5	26	1.7	0.7	33				1.5	0.7	38	7.7	4.0	6	303	109	17									
Lopes *et al.*[21]	Portugal	1600	800	11	1.8	0.5	12	1.9	0.6	20	22.0	5.8	11	2.1	0.6	9	8.2	3.8	4	312	156	24	121	57	14	3.4	1.6	100	8.3	2.7	20
Luhrmann *et al.* [23]	Germany	1530	1320	24																			121	61	16				13.5	4.5	5
Max Rubner-Institut [24]	Germany	2100	1100	9	1.4	0.5	34	1.8	0.7	28	32.7	9.6	3	2.2	0.7	10	5.9	2.5	4	276	93	21	142	70	12	4.4	4.1	91	6.3	6.3	48
Milman *et al.* [25]	Denmark	1253	1113	28	1.0	0.3	73	1.6	0.5	36	24.9	6.6	7	1.2	0.4	60	5.7	3.7	12	288	143	27	53	47	56	5.5	6.9	74	7.6	3.6	33
Mowe *et al.* [26]	Norway	947	449	22	1.4	0.7	39	1.9	0.8	27	16.6	5.6	39										72	48	40	8.0	8.0	60			
Nelson *et al.* [27]	USA																			303	124	20									
Nicolas *et al.* [28]	France	1332	1759	34	1.1	0.3	63	1.7	0.6	31				1.6	0.4	23	9.0	11.0	24	286	115	23	121	71	20				8.0	4.0	31
Ocke *et al.* [29]	Netherlands	1326	808	18	1.1	0.6	57	1.6	0.6	37				2.1	0.8	37	5.7	4.6	17	367	174	17	103	52	20	4.7	3.4	94	15.1	7.8	12
Ortega *et al.* [30]	France	1080	1363	36	1.2	0.3	51	1.5	0.5	43	28.3	6.9	3	1.5	0.4	36	9.0	9.2	20	208	79	46	134	80	18	3.3	3.2	98	5.1	2.3	65
Pedersen *et al.* [31]	Denmark	1293	998	24	1.3	0.4	40	1.7	0.6	31	32.0	9.0	3	1.5	0.5	34				303	119	19	95	52	25	3.9	4.0	94	7.2	3.2	35
Pietinen *et al.* [32]	Finland	836	1097	41	1.3	0.5	42	1.8	0.8	31	30.0	11.0	9	2.0	1.2	28	6.5	6.0	20	243	102	34	92	72	33	9.0	8.2	55	8.7	3.9	24
Posner *et al.* [33] 70-79y	USA	1808	1717	24	1.4	0.8	42																120	100	27						
Posner *et al.* [33] 80+y	USA	1761	1821	26	1.2	0.6	51																106	90	31						
Rothenberg *et al.* [34]	Sweden				1.5	0.1	0	1.9	1.0	31													79	59	37	6.6	3.9	81			
Serra Majem *et al.*[35]	Spain	500	100	84	1.0	0.2	84	1.3	0.3	63	16.4	2.7	30	1.7	0.3	9	3.8	0.6	0	236	47	22	126	50	9	7.0	1.0	100	8.4	2.3	15
Sette *et al.* [36]	Italy	888	851	37	1.0	0.3	74	1.5	0.4	42				2.1	0.6	9	6.5	5.5	18				127	74	18	2.5	2.4	100	13.3	4.5	5
Szponar *et al.* [37]	Poland	1500	3300	39	1.3	0.6	43	1.8	1.0	34				1.9	0.8	23	5.4	9.1	33	255	132	34	73	57	41	4.4	5.9	83	11.5	6.4	20
Toffanello *et al.* [38]	Italy	1347	725	15	0.9	0.3	84	1.6	0.5	34													102	71	28						
USDA *et al.* [39]	USA	738	1067	45	1.7	1.0	32	2.2	1.5	28	24.4	14.7	26	2.1	1.5	29	6.0	7.2	26	207	123	48	90	154	42	5.8	8.8	68	8.2	8.1	39
Zoltick *et al.* [40]	USA																									8.0	7.0	61			
Mean Intake		1273		29	1.3		50	1.7		41	27.1		15	1.8		31	6.4		16	278		29	99		29	5.4		84	9.6		26
SD (mean)		489			0.3			0.4			7.2			0.4			1.5			61			25			2.7			3.0		

RE, retinol equivalent; TE. tocopherol equivalent.

Table S3 Individual study data and % of the population at risk for inadequacy, mineral intakes among older men.

		Calcium (mg/d)			Copper (mg/d)			Iodine (µg/d)			Iron (mg/d)			Magnesium (mg/d)			Phosphorus (mg/d)			Potassium (g/d)		Selenium (µg/d)			Sodium (g/d)		Zinc (mg/d)		
Reference	Country	Mean	SD	%	Mean	SD	%	Mean	SD	%	Mean	SD	%	Mean	SD	%	Mean	SD	%	Mean	SD	Mean	SD	%	Mean	SD	Mean	SD	%
Adamson *et al.* [3]	UK	1081	230	36							18.7	16.7	24																
Bates *et al.* [4]	UK	971	335	53	1.5	0.9	19	223	101	11	11.7	4.6	15	279	98	77				3.1	0.9	59	49	31			10.3	4.8	19
Becker *et al.* [5]	Sweden	1071	406	43							12.1	3.6	8	323	90	62	1571	475	1	3.7	1.1	40	14	36	3.4	1.0	12.0	3.6	5
Boilson *et al.* [6]	Ireland	669	292	87																									
Castetbon *et al.* [7]	France	893	319	63																					4.1	2.3			
Decarli *et al.* [8]	Switzerland	1031	334	46							13.0	3.0	2																
Elmadfa *et al.* [9]	Austria	649	295	88	1.9	0.6	2	188	64	8	10.9	3.4	13	256	70	91	1059	390	6	2.2	0.7				3.4	0.9	9.4	3.0	13
Elmadfa *et al.* [10]	Romania	931	365	57							25.2	7.0	0	291	85	76													
Feart *et al.* [11]	France	924	442	57							13.3	6.2	15	292	81	76	1246	387	2	3.0	0.9						7.0	5.3	43
Fidanza *et al.* [12] 65-69.9y	Italy	635	206	96							18.0	4.7	1												2.3	0.9			
Fidanza *et al.* [12] 70+y	Italy	662	249	91							16.6	5.8	5												2.4	1.0			
Finch *et al.* [13]	UK	764	252	83							10.6	4.8	23																
Gibson [14]	UK	839	290	71							11.1	4.1	16																
Griep *et al.* [15]	Belgium	622	179	98							13.0	2.4	1																
Health Canada *et al.* [16]	Canada	762	1287	57							13.7	11.7	28	318	312	54	1244	1209	26	3.1	2.7				2.9	2.5	10.3	7.8	29
Horwath *et al.* [17]	New-Zealand	847	288	70							11.6	3.0	6	300	84	72				3.1	0.7	34	11	55			9.3	2.4	8
Hulshof *et al.* [18]	Netherlands	1024	414	48	1.1	0.4	16	152	73	24	11.4	3.8	12	284	74	81	1576	472	1	3.8	1.1	48	24	29			10.0	3.3	11
Johansson *et al.* [19]	Norway	861	349	65							12.3	5.6	17	277	69	85													
Konstantinova *et al.* [20]	Norway																												
Lopes *et al.* [21]	Portugal	853	316	68							16.3	4.8	3	312	105	64	1367	379	1	3.8	1.0				3.7	1.0			
Luhrmann *et al.* [23]	Germany	997	361	50							15.2	4.0	2																
Max Rubner-Institut [24]	Germany	970	357	53							13.6	3.9	5	348	102	51				3.5	0.9				3.1	0.9	10.9	3.2	6
Milman *et al.* [25]	Denmark	902	374	60				117	92	43	8.9	2.8	26	276	83	81	1190	349	2	2.6	0.8	39	17	41	2.8	1.0	9.7	2.5	7
Mowe *et al.* [26]	Norway	1211	622	37							13.0	3.8	6																
Nelson *et al.* [27]	USA																												
Nicolas *et al.* [28]	France	984	321	52							15.0	5.0	5														9.0	2.0	7
Ocke *et al.* [29]	Netherlands	1021	312	47	1.2	0.5	16	172.2	55	9	11.4	4.2	15	347	87	52	1545	378	93	3.5	0.8	49	21	25	2.6	0.9	11.1	3.6	8
Ortega *et al.* [30]	France	784	308	76				296	161	11	12.1	3.3	6	234	70	95											9.6	2.9	10
Pedersen *et al.* [31]	Denmark	952	420	55							10.9	3.1	10	308	76	71	1412	448	2	3.6	1.0	44	15	27	3.3	1.1	10.8	3.1	6
Pietinen *et al.* [32]	Finland	1032	521	48	1.4	0.5	8	194	75	11	12.6	5.6	16	379	115	40				3.7	1.1	66	26	12	3.1	1.3	12.3	4.3	7
Posner *et al.* [33] 70-79y	USA	715	477	72																									
Posner *et al.* [33] 80+y	USA	552	312	92																									
Rothenberg *et al.* [34]	Sweden	1057	581	46							17.0	9.8	15																
Serra Majem *et al.* [35]	Spain	757	152	95							11.2	1.3	0	261	50	96	1165	187	0	2.8	0.4				2.3	0.5	7.5	1.0	7
Sette *et al.* [36]	Italy	825	331	70							13.2	3.8	5	295	81	75	1331	332	0	3.3	0.9						12.2	3.2	3
Szponar *et al.* [37]	Poland	580	315	91	1.3	0.4	7	107	40	43	13.8	14.0	31	240	84	90	1257	458	4	3.5	1.3	51	28	28	4.6	1.8	11.0	4.3	12
Toffanello *et al.* [38]	Italy																												
USDA *et al.* [39]	USA	895	768	55	1.3	1.32	32				16.4	12.1	22	290	165	64	1272	689	12	2.7	1.6	102	53	10	3.2	1.6	12.1	7.0	19
Zoltick *et al.* [40]	USA	770	401	72																									
Mean Intake		864		65	1.4		14	181		20	13.7		11	296		73	1326		11	3.2		53		30	3.1		10.3		12
SD (mean)		159			0.2			57			3.1			35			156			0.5		19			0.6		1.5		

Table S4 Individual study data and % of the population at risk for inadequacy, vitamin intakes among older women.

		Vitamin A (µg-RE/d)			Thiamine (mg/d)			Riboflavin (mg/d)			Niacin (mg/d)			Vitamin B_6 (mg/d)			Vitamin B_{12} (µg/d)			Folate (µg/d)			Vitamin C (mg/d)			Vitamin D (µg/d)			Vitamin E (α-TE/d)		
Reference	Country	Mean	SD	%	Mean	SD	%	Mean	SD	%	Mean	SD	%	Mean	SD	%	Mean	SD	%	Mean	SD	%	Mean	SD	%	Mean	SD	%	Mean	SD	%
Adamson *et al.* [3]	UK																						135	70	11						
Bates *et al.* [4]	UK	1309	1135	24	1.7	2.6	37	2.2	3.6	38	32.6	10.9	3	2.5	2.8	30	6.3	5.0	16	266	109	27	97	87	30	4.5	3.8	93			
Becker *et al.* [5]	Sweden	1400	700	10	1.3	0.4	16	1.7	0.5	12	31.9	8.3	1	2.0	0.6	5	7.4	4.1	7	237	81	32	116	68	17	6.1	2.0	97	7.1	2.4	19
Boilson *et al.* [6]	Ireland																4.3	3.3	19	351	128	12	154	92	13	3.8	2.2	100			
Castetbon *et al.* [7]	France																														
Decarli *et al.* [8]	Switzerland	865	349	15	0.8	0.3	62	1.3	0.4	33				0.9	0.2	62							114	49	10						
Elmadfa *et al.* [9]	Austria	1300	1700	32	0.9	0.3	50	1.2	0.4	40	22.7	5.7	3	1.2	0.4	31	4.2	2.4	12	166	62	71	115	60	14	2.4	2.1	100	12.5	4.5	5
Elmadfa *et al.* [10]	Romania	1900	700	2	1.4	0.5	16	2.6	1.3	12													76	40	26						
Feart *et al.* [11]	France	694	3305	48	0.9	0.4	46	1.5	0.8	31				1.3	0.5	27	5.4	10.9	36	257	128	33	82	59	30	1.6	2.9	100	6.1	4.2	40
Fidanza *et al.* [12] 65-69.9y	Italy	857	899	35	0.8	0.3	71	0.9	0.3	68													56	26	41						
Fidanza *et al.* [12] 70+y	Italy	656	733	42	0.7	0.2	83	0.9	0.3	76													58	26	38						
Finch *et al.* [13]	UK																						52	39	48						
Gibson [14]	UK							1.4	0.0	50										211	73	44	61	39	39	2.8	1.9	100			
Griep *et al.* [15]	Belgium	630	210	27	1.0	0.2	33				13.2	3.9	38	1.3									84	55	27						
Health Canada *et al.* [16]	Canada	630	1124	45	1.4	1.5	38	1.5	2.0	42	29.8	30.7	28	1.6	1.5	36	3.5	10.2	42	340	307	32	106	153	36	5.3	15.3	62			
Horwath *et al.* [17]	New-Zealand	1538	605	4	1.1	0.3	24	1.0	0.2	74	25.9	6.8	2	1.3	0.3	22	4.3	2.3	10	227	64	34	121	72	16	2.4	1.3	100			
Hulshof *et al.* [18]	Netherlands	800	500	27	1.1	0.6	37	1.5	0.5	21				1.4	0.4	16				177	75	62	95	53	20	3.6	2.1	100	11.6	6.6	16
Johansson *et al.* [19]	Norway	2300	1300	8	1.8	1.4	26	2.1	1.5	25	20.8	16.1	29										160	87	10	12.9	11.8	40			
Konstantinova *et al.* [20]	Norway				1.3	0.6	26	1.5	0.8	32				1.3	0.6	33	5.5	3.5	12	265	101	26									
Lopes *et al.* [21]	Portugal	1600	800	8	1.6	0.4	4	1.9	0.6	9	20.1	5.1	6	2.0	0.6	5	7.5	4.1	7	290	163	29	118	57	12	3.3	1.5	100	7.7	2.5	14
Luhrmann *et al.* [23]	Germany	1290	1550	31				1.6	0.6	19													117	54	11				12.9	4.8	5
Max Rubner-Institut [24]	Germany	1700	800	7	1.2	0.6	31	1.6	0.8	27	25.9	0.7	0	1.8	0.9	19	4.3	2.0	7	276	93	21	148	81	11	3.4	2.8	99	12.6	6.5	12
Milman *et al.* [25]	Denmark	1416	1478	27	0.9	0.3	45	1.5	0.8	32	21.5	6.0	6	1.1	0.3	40	5.7	6.0	24	319	283	34	58	47	43	4.2	6.4	82	6.2	2.4	31
Mowe *et al.* [26]	Norway	658	338	32	1.4	1.1	32	1.5	0.6	25	11.8	3.1	53										65	39	35	4.9	3.7	92			
Nelson *et al.* [27]	USA																			331	128	15									
Nicolas *et al.* [28]	France	1196	1687	34	1.0	0.3	37	1.6	0.6	20				1.4	0.4	16	8.0	10.0	25	258	105	29	121	69	15				7.0	3.0	25
Ocke *et al.* [29]	Netherlands	808	764	34	1.0	0.5	42	1.3	0.5	19				1.7	0.6	19	4.7	3.8	19	298	125	22	102	51	16	3.5	2.2	100	11.1	5.3	12
Ortega *et al.* [30]	France	898	740	30	1.0	0.3	33	1.4	0.3	16	25.9	6.9	2	1.4	0.4	19	5.7	4.5	17	208	71	46	128	62	10	3.3	4.1	95	5.0	2.0	51
Pedersen *et al.* [31]	Denmark	961	609	22	1.0	0.3	37	1.4	0.4	25	26.0	7.0	2	1.2	0.4	31				279	98	21	115	67	17	3.4	2.8	99	6.6	3.0	30
Pietinen *et al.* [32]	Finland	770	1150	41	1.0	0.4	40	1.5	0.6	25	23.0	7.0	6	1.5	0.7	24	5.2	4.8	21	210	96	46	97	68	24	6.5	4.6	78	7.3	3.2	24
Posner [33] 70-79y	USA	1895	1816	22	1.1	0.7	36																133	111	23						
Posner [33] 80+y	USA	1861	1796	22	1.1	0.6	38																121	91	22						
Rothenberg *et al.* [34]	Sweden				1.3	1.1	35	1.6	0.7	24													88	86	33	5.4	3.2	92			
Serra Majem *et al.* [35]	Spain	400			1.0	0.2	31	1.3	0.2	16	14.4	1.2	2	1.5	0.2	1	3.5	0.5	0	216	28	28	115	38	4	7.0	1.0	100	7.5	1.7	7
Sette *et al.* [36]	Italy	773	466	28	0.9	0.3	56	1.3	0.4	30				1.7	0.5	8	4.4	3.5	20				127	84	18	1.8	1.7	100	10.9	3.7	6
Szponar *et al.* [37]	Poland	1200	2400	39	1.0	0.5	42	1.5	0.8	31				1.5	0.6	20	3.4	5.7	36	213	101	45	68	53	37	2.9	2.9	99	9.3	5.3	21
Toffanello *et al.* [38]	Italy	1080	673	19	0.8	0.4	60	1.5	0.8	31													121	80	19						
USDA *et al.* [39]	USA	612	414	39	1.3	0.9	31	1.8	1.0	26	18.6	13.1	31	1.7	1.5	33	4.2	5.4	30	178	118	57	80	75	35	4.4	3.2	96	6.3	5.9	41
Zoltick *et al.* [40]	USA																									8.4	6.4	60			
Mean Intake		1133		26	1.1		39	1.5		31	22.8		13	1.5		24	5.1		19	253		35	103		23	4.5		91	8.7		21
SD (mean)		472			0.3			0.4			6.0			0.4			1.3			53			29			2.4			2.6		

RE, retinol equivalent; TE. tocopherol equivalent.

Table S5 Individual study data and % of the population at risk for inadequacy, mineral intakes among older women.

		Calcium (mg/d)			Copper (mg/d)			Iodine (µg/d)			Iron (mg/d)			Magnesium (mg/d)			Phosphorus (mg/d)			Potassium (g/d)		Selenium (µg/d)			Sodium (g/d)		Zinc (mg/d)		
Reference	Country	Mean	SD	%	Mean	SD	%	Mean	SD	%	Mean	SD	%	Mean	SD	%	Mean	SD	%	Mean	SD	Mean	SD	%	Mean	SD	Mean	SD	%
Adamson *et al.* [3]	UK	972	203	55							16.1	4.3	1																
Bates *et al.* [4]	UK	848	322	68	1.2	0.8	27	175	72	15	10.4	4.2	15	228	61	73				2.6	0.6	43.0	17.0	22			9.0	5.5	23
Becker *et al.* [5]	Sweden	937	312	58							11.8	4.9	12	350	97	19	1357	394	1	3.3	0.9	37.0	14.0	31	2.9	0.7	10.5	2.9	3
Boilson *et al.* [6]	Ireland	617	228	95																									
Castetbon *et al.* [7]	France	818	326	71																					2.9	1.5			
Decarli *et al.* [8]	Switzerland	828	273	74							10.0	2.0	2																
Elmadfa *et al.* [9]	Austria	693	276	87	1.9	0.5	1	190	60	7	10.3	2.9	7	269	89	48	984	291	3	2.2	0.6				3.2	0.9	8.8	2.5	6
Elmadfa *et al.* [10]	Romania	846	334	68							20.9	6.5	1	341	99	22													
Feart *et al.* [11]	France	829	422	66							9.7	4.9	23	234	76	66	1029	370	6	2.5	0.8						7.7	7.4	36
Fidanza [12] 65-69.9y	Italy	622	238	94							11.5	4.2	10												1.9	0.9			
Fidanza [12] 70+	Italy	609	236	95							10.6	3.0	6												1.6	0.6			
Finch *et al.* [13]	UK	656	261	91							7.9	3.0	26																
Gibson [14]	UK	693	239	90							8.8	3.9	24																
Griep *et al.* [15]	Belgium	586	142	100							11.0	2.3	1																
Health Canada *et al.* [16]	Canada	690	868	64							11.1	10.2	31	268	204	49	1055	971	27	2.6	2.0				2.3	2.5	8.5	10.2	37
Horwath *et al.* [17]	New-Zealand	812	316	72							10.1	2.5	5	277	74	44				2.9	0.7	29.6	9.1	52			8.0	2.0	7
Hulshof *et al.* [18]	Netherlands	959	359	55	1.0	0.3	16	116	53	38	10.1	2.8	7	332	101	25	1338	340	0	3.3	0.8	39.0	15.0	27			8.9	2.5	6
Johansson *et al.* [19]	Norway	776	266	80							10.8	4.6	15	333	93	23													
Konstantinova *et al.* [20]	Norway																												
Lopes *et al.* [21]	Portugal	904	375	60							14.2	4.7	4	354	108	20	1312	414	2	3.4	1.0				3.3	0.9			
Luhrmann *et al.* [23]	Germany	1010	331	49							12.6	3.2	2																
Max Rubner-Institut [24]	Germany	918	342	59							11.4	3.5	6	403	109	10				3.1	0.9				2.4	0.7	8.8	2.8	9
Milman *et al.* [25]	Denmark	895	329	63				88	51	59	7.6	2.3	25	242	65	64	1074	310	2	2.4	0.7	33.0	14.0	42	2.2	0.7	8.6	2.1	4
Mowe *et al.* [26]	Norway	913	485	57							9.1	2.5	11																
Nelson *et al.* [27]	USA																												
Nicolas *et al.* [28]	France	909	322	61							11.0	3.0	5														8.0	2.0	7
Ocke *et al.* [29]	Netherlands	953	374	55	1.0	0.3	16	146	49	17	9.1	2.9	14	288	80	39	1310	387	99	3.0	0.8	42.1	16.3	23	2.1	0.7	9.7	3.3	8
Ortega *et al.* [30]	France	754	216	87				285	116	6	10.1	2.6	5	219	60	78											8.3	2.3	8
Pedersen *et al.* [31]	Denmark	880	337	64							8.8	2.6	14	364	102	17	1164	334	2	3.1	0.8	36.0	12.0	31	2.6	0.8	8.5	2.3	6
Pietinen *et al.* [32]	Finland	900	426	59	1.2	0.5	16	175	78	17	9.9	3.6	14	313	95	31				3.2	0.9	49.0	17.0	13	2.3	0.7	9.4	2.9	6
Posner *et al.* [33] 70-79y	USA	611	393	84																									
Posner *et al.* [33] 80+y	USA	602	408	84																									
Rothenberg *et al.* [34]	Sweden	936	559	55							14.0	10.8	23																
Serra Majem *et al.* [35]	Spain	712	108	100							9.6	1.0	0	285	48	34	1045	112	0	2.6	0.4				1.8	0.4	6.8	0.7	1
Sette *et al.* [36]	Italy	754	290	80							10.0	3.0	9	243	66	63	1117	305	1	2.8	0.8						9.9	2.9	5
Szponar *et al.* [37]	Poland	533	286	95	1.0	0.4	23	97	38	53	9.8	6.2	27	300	100	36	1002	363	6	2.9	1.0	36.0	19.0	38	3.2	1.2	8.4	3.5	17
Toffanello *et al.* [38]	Italy																												
USDA *et al.* [39]	USA	813	292	74	1.1	0.7	28				12.6	8.4	22	243	118	57	1061	417	7	2.3	0.8	81.4	53.9	17	2.6	1.5	9.5	7.2	27
Zoltick *et al.* [40]	USA	848	454	63																									
Mean Intake		795		73	1.2		18	159		26	11.0		12	294		41	1142		12	2.8		42.6		30	2.5		8.7		12
SD (mean)		130			0.3			59			2.5			51			133			0.4		13.9			0.5		0.9		

References

1. Wells GA, Shea B, O'Connell D *et al.* (2011) The Newcastle-Ottawa Scale (NOS) for assessing the quality of nonrandomised studies in meta-analyses. http://www.ohri.ca/programs/clinical_epidemiology/oxford.asp (accessed December 2012)
2. Cochrane Form III for the evaluation of a cohort-study (Formulier III: voor de beoordeling van het cohortonderzoek) (2012) Coding manual for cohort studies. http://dcc.cochrane.org/sites/dcc.cochrane.org/files/uploads/cohort.pdf (accessed November 2012)
3. Adamson AJ, Collerton J, Davies K *et al.* (2009) Nutrition in advanced age: dietary assessment in the Newcastle 85+ study. *Eur J Clin Nutr* **63**, Suppl 1, S6-18.
4. Bates E, Lennox A, Bates C *et al.* (2011) National diet and nutrition survey. Headline results from years 1 and 2 (combined) of the rolling programme (2008/2009 - 2009/2010). https://www.gov.uk/government/uploads/system/uploads/attachment_data/file/216484/dh_128550.pdf (accessed April 2014)
5. Becker W & Pearson M (2002) *Riksmaten 1997–98. Dietary habits and nutrient intake in Sweden*. Uppsala: Livsmedelsverket, National Food Agency.
6. Boilson A, Staines A, Kelleher CC *et al.* (2012) Unmetabolized folic acid prevalence is widespread in the older Irish population despite the lack of a mandatory fortification program. *Am J Clin Nutr* **96**, 613-621.
7. Castetbon K, Vernay M, Malon A *et al.* (2009) Dietary intake, physical activity and nutritional status in adults: the French nutrition and health survey (ENNS, 2006-2007). *Br J Nutr* **102**, 733-743.
8. Decarli B, Dirren H, Schlettwein-Gsell D (1998) Swiss survey in europe on nutrition and the elderly: nutritional status of a Yverdon population aged 74 to 79 years old over a period of four years. *Rev Med Suisse Romande* **118**, 701-707.
9. Elmadfa I, Freisling H, Nowak V *et al.* (2009) *Austrian nutrition report 2008 (Österreichischer ernährungsbericht 2008)*. Wien: Bundesministerium für Gesundheit.
10. Elmadfa I, Meyer A, Nowak V *et al.* (2009) European nutrition and health report 2009. *Ann Nutr Metab* **55**, Suppl 2, S1-40.
11. Feart C, Alles B, Merle B *et al.* (2012) Adherence to a Mediterranean diet and energy, macro-, and micronutrient intakes in older persons. *J Physiol Biochem* **68**, 691-700.
12. Fidanza F, Simonetti MS, Mariani Cucchia L *et al.* (1984) Nutritional status of the elderly. II. Anthropometry, dietary and biochemical data of old pensioners in Perugia at the fifth year follow-up. *Int J Vitam Nutr Res* **54**, 75-90.
13. Finch S, Doyle W, Lowe S *et al.* (1998) *National diet and nutrition survey: People aged 65 years and over. Volume 1: report of the diet and nutrition survey.* London: The Stationery Office.
14. Gibson S (2001) Dietary sugars and micronutrient dilution in normal adults aged 65 years and over. *Public Health Nutr* **4**, 1235-1244.
15. Griep MI, Verleye G, Franck AH *et al.* (1996) Variation in nutrient intake with dental status, age and odour perception. *Eur J Clin Nutr* **50**, 816-825.
16. Health Canada, Statistics Canada (2009) *Canadian community health survey, cycle 2.2, nutrition (2004). Nutrient intakes from food, provincial, regional and national summary data tables, volume 1, 2 and 3.* Ottawa: Health Canada.
17. Horwath CC, Campbell AJ, Busby W (1992) Dietary survey of an elderly New Zealand population. *Nutr Res* **12**, 441-453.
18. Hulshof KFAM, van Staveren WA (1991) The Dutch national food consumption survey: design, methods and first results. *Food Policy* **16**, 257-260.
19. Johansson L, Solvoll K (1999) *Norkost 1997. National food consumption survey among men and women aged 16-79 years. (Landsomfattende kostholdsundersøkelse blant menn og kvinner i alderen 16-79 år)*. Oslo: Statens råd for ernæring og fysisk aktivitet.
20. Konstantinova SV, Tell GS, Vollset SE *et al.* (2008) Dietary patterns, food groups, and nutrients as predictors of plasma choline and betaine in middle-aged and elderly men and women. *Am J Clin Nutr* **88**, 1663-1669.
21. Lopes C, Oliveira A, Santos AC *et al.* (2006) *Food consumption in Porto. (Consumo alimentar no Porto)*. Porto: Department of hygiene and epidemiology.
22. Lührmann PM, Herbert BM, Neuhäuser-Berthold M (2001) Underreporting of energy intake in an elderly German population. *Nutrition (Burbank, Los Angeles County, Calif)* **17**, 912-916.
23. Luhrmann PM, Herbert BM, Neuhauser-Berthold M (2001) Underreporting of energy intake in an elderly German population. *Nutrition* **17**, 912-916.
24. Max rubner-institut (2008) *National consumption study II. Results report part 1. The national survey on nutrition of adolescents and adults. (Nationale verzehrsstudie II. Ergebnisbericht teil 1. Die bundesweite befragung zur ernahrung von jugendlichen und erwachsenen)*. Karlsruhe: Max rubner institut.

25. Milman N, Pedersen AN, Ovesen L *et al.* (2004) Iron status in 358 apparently healthy 80-year-old Danish men and women: relation to food composition and dietary and supplemental iron intake. *Ann Hematol* **83**, 423-429.
26. Mowe M, Bohmer T, Kindt E (1994) Reduced nutritional status in an elderly population (> 70 y) is probable before disease and possibly contributes to the development of disease. *Am J Clin Nutr* **59**, 317-324.
27. Nelson C, Wengreen HJ, Munger RG *et al.* (2009) Dietary folate, vitamin B-12, vitamin B-6 and incident alzheimer's disease: the cahce country memory, health, and aging study. *J Nutr Health Aging* **13**, 899-905.
28. Nicolas AS, Faisant C, Nourhashemi F *et al.* (2001) Nutrient adequacy of dietary intake in a healthy elderly French population. *Eur J Ger* **3**, 140-145.
29. Ocke MC , Buursma-Rethans EJM, de Boer EJ *et al.* (2013) *Diet of community-dwelling older adults: Dutch national food consumption survey older adults 2010-2012*. Bilthoven: National institute for public health, sport and the environment.
30. Ortega RM, Requejo AM, Andres P *et al.* (1997) Dietary intake and cognitive function in a group of elderly people. *Am J Clin Nutr* **66**, 803-809.
31. Pedersen AN, Fagt S, Groth M *et al.* (2010) *Dietary habits in Denmark 2003-2008. Main results*. Søborg: DTU food, National food institute.
32. Pietinen P, Paturi M, Reinivuo H *et al.* (2010) FINDIET 2007 survey: energy and nutrient intakes. *Public Health Nutr* **13**, 920-924.
33. Posner BM, Jette A, Smigelski C *et al.* (1994) Nutritional risk in New England elders. *J Gerontol* **49**, M123-132.
34. Rothenberg E, Bosaeus I, Steen B (1996) Food habits and nutrient intake in three 70-year-old free-living populations in Gothenburg, Sweden. A 22-year cohort study. *Scand J Nutr* **40**, 104-110.
35. Serra Majem L, Ribas Barba L, Salvador Castell G *et al.* (2006) *Assessment of nutritional status of the Catalan population 2002-2003. Evolution of food habits and consumption of food and nutrients in Catalonia (1992-2003). (Avaluació de l'estat nutricional de la població catalana 2002-2003. Evolució dels hàbits alimentaris i dels consum d'aliments i nutrients a Catalunya (1992-2003))*. Barcelona: Departament de salut, Generalitat de Catalunya.
36. Sette S, Le Donne C, Piccinelli R *et al.* (2011) The third Italian national food consumption survey, INRAN-SCAI 2005-06-part 1: nutrient intakes in Italy. *Nutr Metab Cardiovasc Dis* **21**, 922-932.
37. Szponar L, Sekula W, Nelson M *et al.* (2001) The household food consumption and anthropometric survey in poland. *Public Health Nutr* **4**, 1183-1186.
38. Toffanello ED, Inelmen EM, Minicuci N *et al.* (2011) Ten-year trends in vitamin intake in free-living healthy elderly people: the risk of subclinical malnutrition. *J Nutr Health Aging* **15**, 99-103.
39. U.S. department of agriculture, Agricultural research service (2012) Nutrient intakes from food: Mean amounts consumed per individual, by gender and age, what we eat in America, NHANES 2009-2010. www.ars.usda.gov/ba/bhnrc/fsrg. (accessed April 2014)
40. Zoltick ES, Sahni S, McLean RR *et al.* (2011) Dietary protein intake and subsequent falls in older men and women: the Framingham study. *J Nutr Health Aging* **15**, 147-152.
